# A Genome-Wide Identification and Analysis of the Basic Helix-Loop-Helix Transcription Factors in Brown Planthopper, *Nilaparvata lugens*

**DOI:** 10.3390/genes7110100

**Published:** 2016-11-18

**Authors:** Pin-Jun Wan, San-Yue Yuan, Wei-Xia Wang, Xu Chen, Feng-Xiang Lai, Qiang Fu

**Affiliations:** State Key Laboratory of Rice Biology, China National Rice Research Institute, Hangzhou 310006, China; wanpinjun@caas.cn (P.-J.W.); yuansanyue@163.com (S.-Y.Y.); weixwang74@126.com (W.-X.W.); 2014102079@njau.edu.cn (X.C.); laifengxiang@caas.cn (F.-X.L.)

**Keywords:** basic helix-loop-helix, *Nilaparvata lugens*, phylogenetic analysis, ortholog family

## Abstract

The basic helix-loop-helix (bHLH) transcription factors in insects play essential roles in multiple developmental processes including neurogenesis, sterol metabolism, circadian rhythms, organogenesis and formation of olfactory sensory neurons. The identification and function analysis of bHLH family members of the most destructive insect pest of rice, *Nilaparvata lugens*, may provide novel tools for pest management. Here, a genome-wide survey for *bHLH* sequences identified 60 *bHLH* sequences (*NlbHLH*s) encoded in the draft genome of *N. lugens*. Phylogenetic analysis of the bHLH domains successfully classified these genes into 40 *bHLH* families in group A (25), B (14), C (10), D (1), E (8) and F (2). The number of *NlbHLHs* with introns is higher than many other insect species, and the average intron length is shorter than those of *Acyrthosiphon pisum*. High number of ortholog families of *NlbHLHs* was found suggesting functional conversation for these proteins. Compared to other insect species studied, *N. lugens* has the highest number of bHLH members. Furthermore, gene duplication events of *SREBP*, *Kn(col)*, *Tap*, *Delilah*, *Sim*, *Ato* and *Crp* were found in *N. lugen*s. In addition, a putative full set of *NlbHLH* genes is defined and compared with another insect species. Thus, our classification of these *NlbHLH* members provides a platform for further investigations of bHLH protein functions in the regulation of *N. lugens*, and of insects in general.

## 1. Introduction

Basic helix-loop-helix (bHLH) proteins are the largest superfamily of transcription factors characterized by a bHLH signature domain for DNA binding. This domain consists of approximately 60 amino acids of two functionally distinctive regions. The basic region locates at the N-terminal end of the domain including ~15 amino acids with a high number of basic residues. The canonical core DNA sequence motif recognized by bHLH is a consensus hexanucleotide sequence known as E-box (5’-CANNTG-3’). E-boxes can be divided into several types based on the identity of two central bases in the sequence. The most common type is the palindromic E-box (5’-CACGTG-3’). Within the basic region of bHLH, certain conserved amino acids serve to identify the core consensus site, whereas other residues in the domain dictate specificity for a given type of E-box [1]. In addition, the nucleotides flanking the hexanucleotide core have been shown to play a role in DNA binding specificity [2,3], and there is evidence that a residue loop outside the core domain plays a critical role in sequence-specific DNA binding through elements that lie outside of the core recognition sequence [4].

The first *bHLH* gene was reported in human in 1988 [5]. To date, with the sequencing of several insect genomes, a large number of *bHLH* family members have been identified in Insecta. Estimations between 48 and 59 putative *bHLH* genes were reported for *Pediculus humanus* corporis [6], *Acyrthosiphon pisum* [7], *Nasonia vitripennis* [8], *Harpegnathos saltator* [9], *Apis mellifera* [10], *Tribolium castaneum* [11], *Leptinotarsa decemlineata* [12], *Bombyx mori* [13], *Anopheles gambiae*, *Aedes aegypti*, *Culex quinquefasciatus* [14] and *Drosophila melanogaster* [15,16,17]. Based on phylogenetic analyses, bHLH proteins have been classified into six main groups that are designated as A, B, C, D, E and F [8,9,10,11,13,14,15,16,17]. This classification exemplifies functional architecture, evolutionary origin, DNA binding specificities and functional activities [8,9,18,19,20] that will be described later.

*Nilaparvata lugens* Stål (Hemiptera, Delphacidae) is a monophagous, phloem-feeding herbivore of rice that causes serious damage. The sequencing of *N. lugens* genome aids the identification of genes that are involved in molting, reproduction and wing development as potential targets for RNA-interference-based management [21,22,23]. bHLHs, as important transcription factors, could be effective targets of RNA interference (RNAi). Although *N. lugens* bHLHs are the focus of several recent publications, the genes have not yet been systematically studied and categorized [24,25,26].

To precisely characterize the bHLHs in *N. lugens*, we systematically analyzed candidate genes from the fully sequenced genome using a known criterion defining the signature bHLH domain. Moreover, we evaluated the phylogenetic relationships among these proteins and those from other organisms, examined the chromosomal distribution and structure diversity of the bHLH domain, and predicted structural and functional activities from the encoded sequences.

## 2. Materials and Methods

### 2.1. Insect Rearing

The *N. lugens* colony used in this work was established from a field collection near the campus of China National Rice Research Institute more than 20 years ago. The colony was maintained on rice (*Oryza sativa*) variety Taichung Native 1 (TN1, a *N. lugens* susceptible variety) in an insectary under controlled conditions of 28 ± 1 °C, 80% ± 10% relative humidity and a 16 h light/8 h dark photoperiod.

### 2.2. *bHLH* Sequence Identification from N. lugens

Sequences of bHLH members from *A. pisum*, *N. vitripennis*, *H. saltator*, *A. mellifera*, *T. castaneum*, *B. mori*, *A. gambiae*, *D. melanogaster*, *Homo sapiens* and *Arabidopsis thaliana*, and their bHLH motifs were obtained from the publicly available genome sequences in Ensembl (Release 86, WTSI/EMBL-EBI, Hinxton, Cambridgeshire, United Kingdom). Each sequence was used as a query to blast search against *N. lugens* genome (version 1, GCA_000757685.1) [21]. The probability of a sequence with significant similarity (e-value) was set at ≤1 to detect all possible genomic hits. Each hit was extended by approximately 10,000 bp (base pairs) upstream and downstream to ensure full-length coverage of the genes. The extended DNA sequences were then downloaded. Genes within the downloaded sequences were predicted by GenScan v.1.0 (Chris Burge, Palo Alto, California, United States) [27], Augustus v.2.5 (Mario Stanke, Gottingen, Niedersachsen, Germany ) [28], FGENESH v.1.6 (Victor Solovyev, Egham, Surrey, United Kingdom) [29] and Exonerate v.2.2 (Guy St C Slater, Hinxton, Cambridgeshire, United Kingdom) [30]. The query sequences also were blast searched (TBLASTN, e-value ≤ 0.001) against *N. lugens* official gene set (kindly provided by Professor Chuanxi Zhang of Zhejiang University) and transcriptome data (SRR1187936) [24,31]. Redundant sequences were manually identified and purposely discarded to keep only one sequence with the same scaffold number, reading frames and coding regions. The sequences were further screened using BLASTX (e-value < 0.00001) against the NCBI non-redundant (nr) database to confirm their *bHLH* identity. The predicted proteins of the screened sequences were subjected to a Pfam protein domain database search [32] using a threshold value of 0.00001. bHLH-like proteins were examined for amino acid residues at 19 conserved sites [2] by manual inspection. The sequences that meet the requirements described by Liu et al. (2012) were considered as potential *N. lugens bHLH*s (*NlbHLH*s).

### 2.3. Multiple Sequence Alignments and Phylogenetic Analysis

Multiple sequence alignments of all the potential bHLH proteins were performed using ClustalW v. 2.1 (EMBL-EBI, Hinxton, Cambridgeshire, United Kingdom) [33] with manual inspection. The alignments were used to construct phylogenetic trees by neighbor-joining (NJ), maximum parsimony (MP), maximum-likelihood (ML) and Bayesian phylogenies using MEGA v.6 (Koichiro Tamura, Hachioji, Tokyo, Japan), PAUP v.4.0 Beta 10 (David Swofford, Sunderland, Massachusetts, United States), RAxML v.8 (Alexandros Stamatakis, Heidelberg, Baden-Württemberg, Germany) [34] and MrBayes v.3.2 (Ronquist and Huelsenbeck, Norbyv. 18D, SE-752 36 Uppsala, Sweden) [35], respectively. Default parameter values of the NJ, ML and MP analyses were used, except for the LG amino acid substitution model with a gamma distribution for among-site rate variation in ML analysis. The reliabilities of NJ, MP and ML tree topology were evaluated by bootstrapping a sample of 1000 replicates. For the Bayesian analysis, the alignment was analyzed using both mixed models that model substitutions as a mixture of many empirical amino-acid substitution matrices, and a LG + γ model for amino acid data. All other parameters such as priors, proposal mechanisms and chain settings were defaults. All sets of chains were performed for 4 million generations, sampled every 100 generations, with 2 million generations discarded as burn-ins. Convergence was confirmed by visual comparison of the likelihoods of two chains in each run, and by using the standard deviation of split frequencies and potential scale reduction factors reported by the software. The best available amino acid substitution model (LG) with a gamma distribution for among-site rate variation used in phylogenetic analysis was estimated by ProtTest v.3 (Diego Darriba, Vigo, Galiza, Spain) under the Akaike information criterion [36]. The ingroup phylogenetic analysis was performed using Liu et al. (2012) described methods with sequence alignments of *NlbHLH* and *DmbHLH* motifs, and the analysis was used to name each *NlbHLH*.

### 2.4. Domain Prediction

The predictions of protein domain architectures were performed to further ascertain the reliability of the retrieved motifs and to examine whether the full-length protein sequences contain additional characteristic domains. Tools available online including Simple Modular Architecture Research Tool (SMART, http://smart.embl-heidelberg.de/) [37], Conserved Domain Architecture Retrieval Tool (CDART, https://www.ncbi.nlm.nih.gov) [38] and PROSITE (http://prosite.expasy.org/) [39] were used.

### 2.5. Molecular Cloning

In order to get transcriptional evidence of the genes, reverse transcription polymerase chain reaction (RT-PCR) was performed to authenticate the sequences of genes or fragments. Total RNA was extracted from eggs, first-instar through fifth-instar nymphs, and newly emerged adults (within 24 h after molting) using the Trizol Reagent (Invitrogen, Shanghai, China) according to the manufacturer’s instructions. These total RNA samples were pooled. The concentration and purity of the pooled sample were measured with the NanoDrop 1000 spectrophotometer (Thermo Fisher Scientific, Rockford, IL, USA) and the integrity was checked by agarose gel electrophoresis. One microgram (μg) of the total RNA was reverse transcribed to cDNA using the ReverTra Ace qPCR RT Kit (Toyobo Co. Ltd., Osaka, Japan). The cDNA was used to perform polymerase chain reaction (PCR) to verify the candidate *NlbHLH*s using primers listed in Table 1. The PCR product was sequenced on the Applied Biosystems 3730 automated sequencer (Foster City, CA, USA) from both directions (Additional file 2). The sequences were aligned with *N. lugens* genome to show their identities.

## 3. Results and Discussion

### 3.1. Identification of *bHLH* Members in *N. lugens*

**Figure 1:**
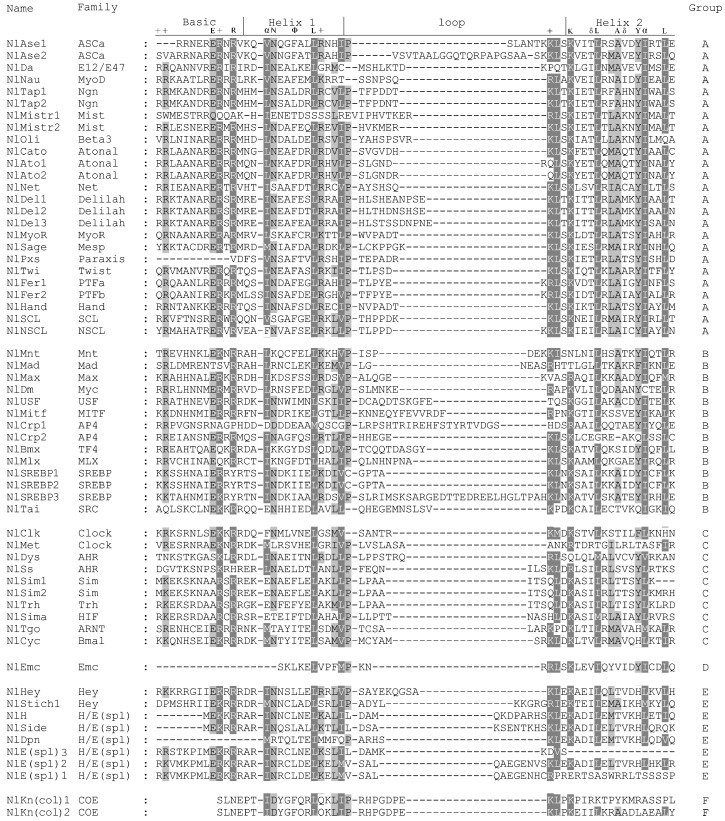
Multiple sequence alignment of basic helix-loop-helix (bHLH) motifs of the 60 *Nilaparvata lugens* basic helix-loop-helix (*NlbHLH*) sequences. The scheme at top illustrates the element of the predicted model and the boundaries of the basic, helix 1, a loop and helix 2 regions within the bHLH domain following that of Atchley et al. (1999) and Ferre-D’Amare et al. (1993), respectively. The dark gray shades indicate identical residues. The light gray shade indicates conserved residues. Hyphens denote gaps. The family names and high-order groups have been organized according to Table 1 of Ledent et al. (2002).

**Figure 2:**
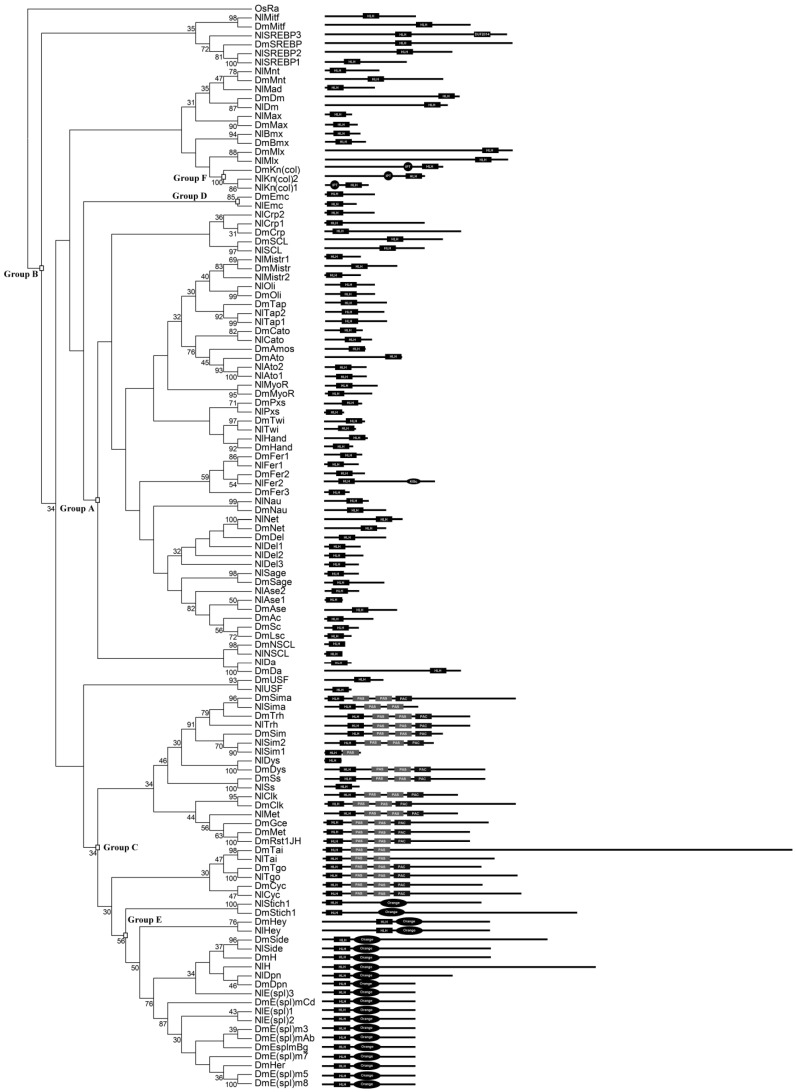
The phylogenetic tree and architecture of 60 NlbHLH members with 59 *D. melanogaster* bHLH members. The left panel is a maximum-likelihood (ML) tree that summarizes the evolutionary relationship between the NlbHLHs and *Drosophila melanogaster* basic helix-loop-helix (*Dmb*HLHs), which has been rooted using OsRa (a rice bHLH motif sequence of R family) as outgroup. This tree is based on a multiple alignment that includes 59 DmbHLH and 60 NlbHLH members. For simplicity, branch lengths of the tree are not proportional to distances between sequences. Only bootstrap values more than 30 are shown. The higher-order group labels are in accordance with Ledent et al. (2002). The right panel is the architecture of HLH and additional domains detected by SMART, CDART and PROSITE, shown by blocks named as HLH, DUF2014, IPT, PAS, PAC, KISc and Orange.

Initially, annotation of the draft *N. lugens* genome (version 1, GCA_000757685.1) and transcriptome (SRR1187936) identified 62 domain-containing *bHLH* genes or gene fragments. These candidate genes were further inspected using blast searches (BLASTX, e-value < 0.00001), intron analysis, manual inspection against the 19 conserved amino acid sites, and sequence alignment. This resulted in 60 unique *bHLH* candidates (*NlbHLHs*). Out of these *NlbHLH*s genes, 48 and 12 were from *N. lugens* official gene sets and *N. lugens* transcriptome, respectively. The alignments of the 60 NlbHLH members were shown in Figure 1. Furthermore, the ML phylogenetic tree (Figure 2) generated with amino acids of the 60 NlbHLH motifs, and 59 DmbHLH motifs were used for their categorization (See Supplementary Figure S1 for NJ, MP and Bayesian tree). This data revealed that 25, 14, 10, 1, 8 and 2 NlbHLH members belong to group A, B, C, D, E and F, respectively. These members possess the basic, helix 1, a loop and helix 2 regions, except for NlPxs, NlEmc, NlH, NlSide, NlSim1, NlDpn and NlE(spl)3 where the basic region or helix 2 was completely or partially missing. The missing regions may reflect the truncated functional roles of these proteins. Additionally, NlFer1 and NlMist1 have one additional amino acid (S or V) in helix 1 or the loop region, respectively. This amino acid creates an additional gap among aligned NlbHLH motifs (Figure 1), indicating certain differences between *N. lugens* and another insect species. In contrast, sites 21 and 64 of the bHLH motif are highly conserved among all NlbHLH motifs (Figure 1). Of these conserved sites, the 19 sites were the most conserved ones in the basic, helix 1, loop and helix 2 regions, as the element of the predicted model [2]. Phylogenetic analysis showed that two or three members of each SREBP, Mnt, COE, AP4, Mist, Ngn, Atonal, Delihah, ASCa, Sim and H/E(spl) family formed a monophyletic clade with that from *D. melanogaster* with high or moderate statistical support (Figure 2). This may suggest relatively recent duplications that were specific to *N. lugens*. Functional redundancy due to gene duplications is a common feature of many biological systems. Feedback between redundant copies may serve as an information processing element that facilitates signal transduction and the control of gene expression [40]. Since the functional roles of bHLH members in *D. melanogaster* have been well studied, we adopted their nomenclature for structural and functional comparison, along with the bootstrap supports provided by the ingroup phylogenetic analyses (Table 2). In the case where one *DmbHLH* sequence has two or more *N. lugens* homologs, they were numbered “1”, “2”, “3”, etc.

### 3.2. Identification of Orthologous Families

**Figure 3:**
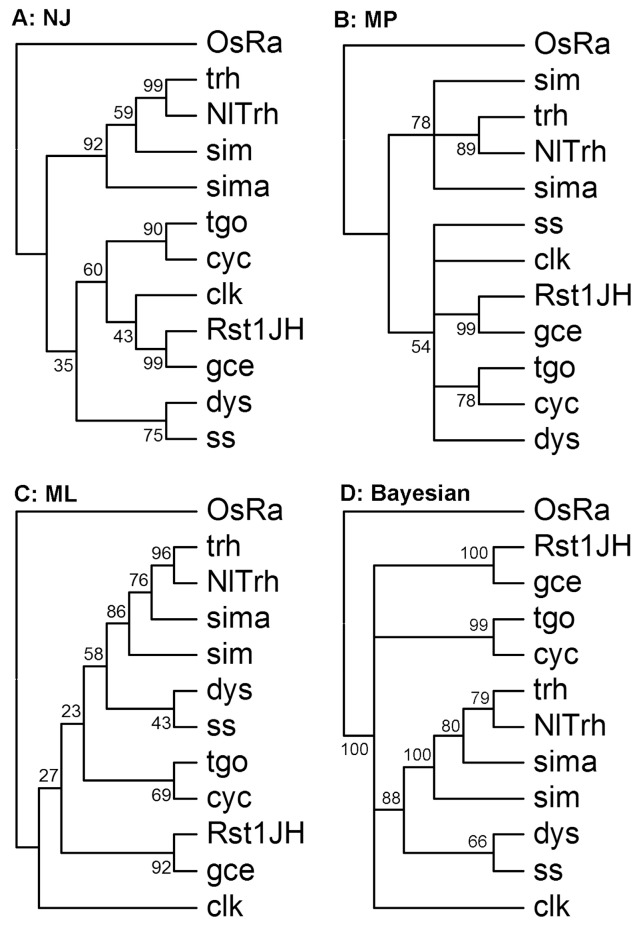
Ingroup phylogenetic analyses of *NlTrh*. (**A**–**D**) are NJ, MP, ML and Bayesian trees, respectively, constructed with one *N. lugens* bHLH member (*NlTrh*) and ten group C bHLH members from *D. melanogaster*. In all the trees, *OsRa* was used as outgroup.

Ingroup phylogenetic analysis of bHLH members has been widely used to define evolutionary conserved groups of orthologs [9]. Previous studies have used monophyletic groups as a standard to define bHLH families of orthologs. A monophyletic group includes members of a known family of different phylogenetic algorithms with statistical support values greater than 50 [9,15,20,41]. Accordingly, we defined evolutionary conserved groups of orthologs according to the ingroup phylogenetic analysis of each NlbHLH member. As an example, Figure 3 shows the NJ, MP, ML and Bayesian inference trees constructed with one NlbHLH member (*trachealess*, *NlTrh*) and 10 group C members from *D. melanogaster*. *NlTrh* formed monophyletic clade with *trh* of *D. melanogaster* with statistical support values of 99, 89, 96 and 79 in NJ, MP, ML and Bayesian inference trees, respectively. *NlTrh* was therefore considered as an ortholog of *D*. *melanogaster trh*. The ingroup phylogenetic analysis was performed to each of the identified *Nl*bHLH members. The statistical support values of the constructed NJ, MP, ML and Bayesian trees were listed in Table 2. The majority of these bHLHs could be clearly assigned to the families according to statistical support values of the ingroup phylogenetic trees. Five NlbHLHs [*NlMad*, *NlH*, *NlE*(*spl*)*1*, *NlE*(*spl*)*2*, *NlE*(*spl*)*3*] could not be confidently assigned by our phylogenetic analysis with DmbHLHs. They were analyzed with *A. pisum* bHLHs (ApbHLH) using the same method mentioned above.

Table 2 shows that orthologs of NlbHLHs with *D*. *melanogaster* or *A. pisum* bHLHs could be grouped into the following categories. Firstly, among all the 60 NlbHLH members, 54 bHLH members had statistical support values of 50 to 100 in the constructed NJ, MP, ML and Bayesian trees. They are *NlAse1*, *NlAse2*, *NlDa*, *NlNau*, *NlTap1*, *NlTap2*, *NlMistr1*, *NlMistr2*, *NlOli*, *NlAto1*, *NlAto2*, *NlNet*, *NlMyoR*, *NlSage*, *NlPxs*, *NlTwi*, *NlFer1*, *NlFer2*, *NlHand*, *NlSCL*, *NlNSCL*, *NlDel1*, *NlDel2*, *NlDel3*, *NlMnt*, *NlMax*, *NlDm*, *NlUSF*, *NlMitif*, *NlCrp1*, *NlCrp2*, *NlBmx*, *NlMlx*, *NlSREBP1*, *NlSREBP2*, *NlSREBP3*, *NlTai*, *NlClk*, *NlDys*, *NlSs*, *NlSim1*, *NlSim2*, *NlTrh*, *NlSima*, *NlTgo*, *NlCyc*, *NlMet*, *NlEmc*, *NlHey*, *NlStich1*, *NlSide*, *NlDpn*, *NlKn(col)1*, and *NlKn(col)2*. Since these statistical support values were greater than the set criterion (50), the genes are assigned as the corresponding *D. melanogaster* homologs (Table 3).

Secondly, one bHLH member, *NlCato*, had statistical support value of 37 in the NJ tree. Nevertheless, it formed a monophyletic clade with the same DmbHLH counterpart in MP, ML and Bayesian trees with statistical support values of 97, 78 and 98, respectively. Consequently, we assigned it to a defined ortholog family according to the three trees with statistical support values of greater than 50.

Thirdly, one bHLH member, *NlDpn*, formed a monophyletic clade in the NJ tree with a statistical support value of 61. A statistical support value of 21 for a monophyletic clade was found in ML, but formed no monophyletic group in MP and Bayesian trees (marked with n/m in Table 2). *NlDpn* forms similar monophyletic group with *DmDpn* and with *A. pisum Dpn* (with statistical support value of 64 and 29 in NJ and ML, respectively). Albeit with insufficient statistical support, we tentatively defined ortholog for *NlDpn* to the correspondent *D. melanogaster dpn*. Obviously, this classification is arbitrary and should be modified if new data becomes available. The phylogenetic divergence of bHLH motif sequences between *N. lugens* and *D. melanogaster* or *A. pisum* probably implies that these insect species evolved in quite different circumstances.

Finally, 5 members named as *NlMad*, *NlH*, *NlE(spl)1*, *NlE(spl)2* and *NlE(spl)3* did not have sufficient bootstrap support in forming a monophyletic clade with any single *D. melanogaster* homolog in all four phylogenetic trees. They were categorized through constructing phylogenetic trees with ApbHLH family members. Four members, namely *NlMad*, *NlH*, *NlE(spl)1* and *NlE(spl)3*, were identified with sufficient confidence (statistical support values > 50) in all the constructed trees. The remaining one member, *NlE(spl)2*, did not form a monophyletic clade with that of *A. pisum*, and was categorized as a *N. lugens* specific clade.

Besides phylogenetic analyses, structure predictions of these *Nl*bHLH proteins were performed. Through predictions by SMART, CDART and PROSITE using the protein sequences of the identified NlbHLH members (Figure 2), we found that: (a) Among members of group C, 6 sequences (NlSim2, NlTrh, NlTgo, NlClk, NlCyc and NlMet) contain one bHLH, one PAC (Motif C-terminal to PAS motifs) [42], and two PAS (Prt-Arnt-Sim) domains. NlTai and NlSima have one bHLH and two PAS domains, respectively. NlSim1 has one bHLH and one PAS domains. The remaining two (NlDys and NlSs) only have the bHLH domain. (b) For group E, all NlbHLHs have bHLH and Orange domains (this domain confers specificity among members of the Hairy/E(spl) family). (c) The two members of group F, NlKn(col)1 and NlKn(col)1, have a IPT domain and a bHLH domain. (d) For group A, NlFer2 has one bHLH domain and KISc domain. The remaining ones only have bHLH domains. (e) NlSREBP3 of group B has one bHLH domain and one DUF2014 domain, whereas the remaining ones only have bHLH domains. (f) The group D member, NlEmc, was predicted to have bHLH domains only. To sum up, these results are consistent with the previous reports of bHLH [9,43,44,45]. It is conceivable that these common domain configurations confer particular protein functions across species [15].

### 3.3. Genomic Distribution of *N. lugens bHLH* Genes

**Figure 4:**
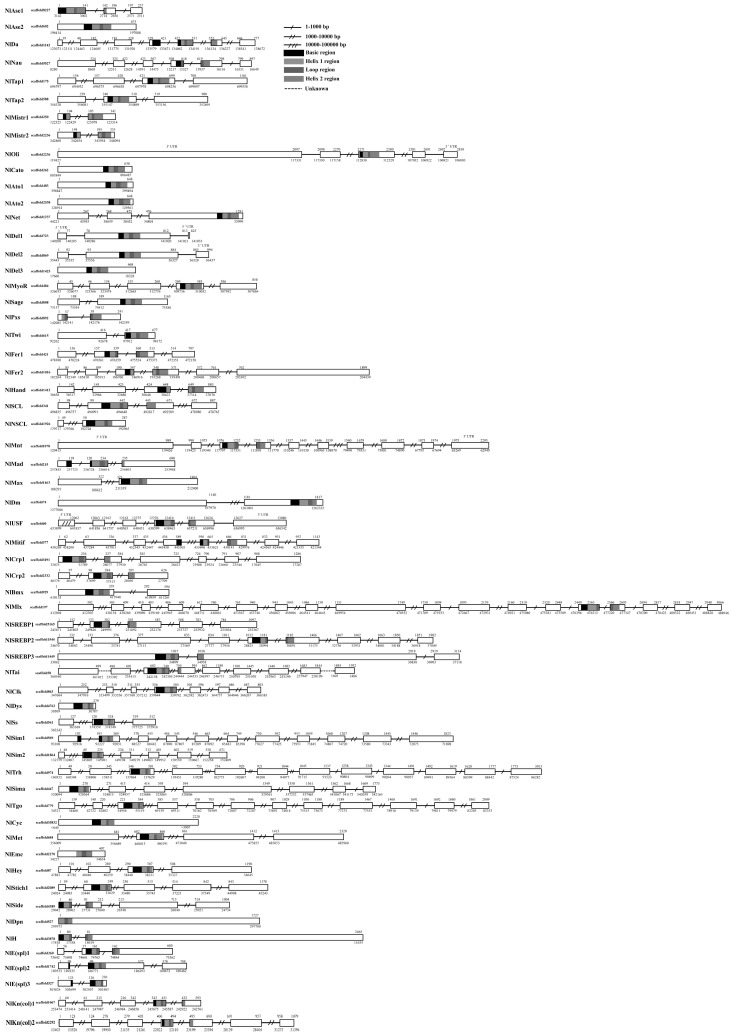
The exon–intron structure of each *NlbHLH* genes in the *N. lugens* genome. Black and white boxes represent exons and introns, respectively. The basic, helix 1, loop and helix 2 regions are shaded in black, respectively. The sites of cDNA and scaffolds are indicated above and below, respectively.

The positions of the 60 *NlbHLH*s in chromosome scaffolds are shown in Figure 4. These *NlbHLH* genes were mapped to 59 *N. lugens* scaffolds. Among these scaffolds, scaffold527 was mapped by two *bHLH* genes, *NlDpn* and *NlE(spl)3*, whereas each of other scaffolds was mapped by one *bHLH* gene. The locations of *NlbHLH* genes on chromosome scaffolds are inconsistent with the hypothetical duplication history of the phylogenetic tree, such as *NlSREBP1* and *NlSREBP1*, *NlKn(col)1* and *NlKn(col)2*, *NlTap1* and *NlTap2*, *NlAto1* and *NlAto2*, etc. This contradiction may be due to the draft genome lacking chromosome-level genome assembly [21].

### 3.4. Intron–Exon Structure of *N. lugens bHLH* Genes

The length of coding regions and exon–intron length are shown in Figure 4. There are eleven intronless genes, and 49 genes having at least one intron. A total of 195 introns were identified with the average intron number of 4.0 per gene. Among these introns, 152 introns are >1000 bp in length (the longest intron is 1,155,031 bp), and the remaining ones are <1000 bp in length (the shortest intron is 35 bp). Intron analysis shows that 29 *NlbHLH* members have introns in the coding regions of their bHLH motifs. It should be noted that: (a) coding regions of 26 *NlbHLH* motifs have one intron, and three motifs have introns in the basic region, five have introns in the helix 1 region, ten have introns in the loop region, and eight have introns in the helix 2 region; and (b) coding regions of three *NlbHLH* motifs have two introns, of which two have introns in the basic and helix 2 regions, and the remaining one has introns in the basic and loop regions. Thus, coding regions of these 29 *NlbHLH* motifs have a total of 32 introns. In addition, one *NlbHLH* (*NlTai*) locates on three separate scaffolds in the genome (Figure 4). In coding regions of *NlbHLH* motifs, the longest intron is 1,155,031 bp, the shortest one is only 35 bp, and the average is 2282 bp. In comparison, *A. pisum*, *D. melanogaster*, *A. aegypti*, *A. gambiae*, *C. quinquefasciatus*, *B. mori*, *A. mellifera*, *N. vitripennis* and *H. saltator* have 26, 18, 24, 22, 19, 12, 9, 22 and 22 *bHLH* members having introns in the coding regions of their *bHLH* motifs, and the total number of introns identified is 34, 20, 30, 26, 23, 12, 9, 27 and 26 with the longest one of 30,718, 11,845, 315,344, 37,485, 8734, 7083, 4460, 174,325 and 7943 bp, the shortest one of 62, 57, 42, 45, 56, 82, 72, 77 and 82, and the average length of 4193, 1082, 15,622, 2024, 1590, 1352, 1326 11,716 and 1391 bp, respectively [7,8,9,10,13,14].

In summary, the number of *NlbHLH*s having introns is higher than that of many other insect species. Moreover, *NlbHLH*s not only have the shortest length intron, but also have longer length introns compared to most studied species (except for *A. aegypti* and *N. vitripennis*). The higher intron-density of *NlbHLH* genes than those of many other insects indicates that *N. lugens* either gained introns at a faster rate or lost introns at a slower rate than others [46]. Previously hypothesized mechanisms of intron gains mainly involve intron transposition [47], transposon insertion [48], tandem genomic duplications [49], intron transfer [50], insertion of a Group II intron [47], intron gain during double strand break repair [51] and intronization [52,53]. Hypothesized mechanisms of intron loss include reverse transcriptase-mediated intron loss [54], meiotic recombination [46] and genomic deletions [55]. Notably, *N. lugens* genome contains a high level of specific transposable element (TE) with larger fraction than that in the *A. pisum*, contributing to the large genome size of *N. lugens* [56]. We speculate that there may be a relationship between the formation of introns in *NlbHLH*s and TEs. Nevertheless, the mechanism of high intron-dense *NlbHLH*s (growing faster or losing slower) needs further investigations.

### 3.5. Molecular Cloning and Predicted Function of *N. lugens bHLHs*

Transcription evidence by RT-PCR and/or EST are widely used for understanding gene functions, e.g., in *N. vitripennis*, *A. aegypti*, *A. gambiae*, *C. quinquefasciatus*, *L. decemlineata*, and *H. saltator*. The transcriptional evidence of 47 *NlbHLHs* (78%) was obtained by both RT-PCR and EST, and the remaining ones were only supported by EST (Table 2). Although RT-PCR as direct evidence is used to support transcription, positive results may not be obtained due to specific temporal and spatial expression patterns or other factors that negatively affect the performance of PCR. Thus, EST as indirect evidence is an additional option to support. We believe that EST supported *NlbHLHs* could denote their highly specific patterns in *N. lugens*. Sequence alignments show that each cDNA and EST exhibited perfect identity with the *N. lugens* genome. As the comparison of cDNA/EST and genome shows, all presumed exon–intron structures are correctly predicted (Addition file 2). Meanwhile, the results support that NlbHLHs play similar functional roles in *N. lugens* as in other insects. Of these NlbHLHs, there are 25 members in group A. The group A proteins bind the E-box variant CACCTG or CAGCTG [20]. This group include proteins such as 48-related-1/Fer1, 48-related-2/Fer2, PTFa/Fer3, ASCa, ASCb, ASCc, amber, Atonal 2, Beta3, Delilah, E12/E47, Hand, Mesp, Mist, MyoD, MyoRa, MyoRb, Net, NeuroD, Neurogenin, NSCL, Oligo, paraxis, peridot, SCL and Twist families [15,57]. These proteins mainly regulate neurogenesis, myogenesis and mesoderm formation [58,59,60,61,62,63]. Our analysis shows that most of NlbHLH members exhibit 1:1 orthology with *D. melanogaster*, suggesting functional conservation.

There are 14 members of NlbHLHs in group B. Group B members recognize and bind G-box (CACGTG or CATGTTG). This group is represented by Figα, Myc, Mnt, Mad, Max, USF, MITF, SRC, SREBP, AP4 and TF4 [15]. The members in this group are mainly involved in cell proliferation/differentiation, sterol metabolism and adipocyte formation, and expression of glucose-responsive genes [9,64,65,66]. We found that the members of SRC, Myc, Mnt, Max, USF, MITF, MLX and AF4 showed 1:1 orthology with *D. melanogaster*. Furthermore, NlbHLHs have more members in SREBP and AP4 families than that of *D. melanogaster*, which could suggest divergent functions of these NlbHLHs.

Ten members of NlbHLHs (NlClk, NlDys, NlSs, NlSim1, NlSim2, NlTrh, NlSima, NlTgo, NlCyc and NlMet) are in group C. Group C is formed by bHLH proteins that have one or two PAS domains in addition to the bHLH motif, and bind to non-E-box (NACGTG or NGCGTG) core sequences [20]. The HLH families of Group C include circadian locomotor output cycles kaput (clock), aryl hydrocarbon receptor (AHR), single-minded (Sim), trachealess (Trh), hypoxia-inducible factor (HIF), aryl hydrocarbon receptor nuclear translocator (ARNT), brain and muscle ARNT-like (Bmal) and methoprene-tolerant (Met). They are responsible for the regulation of multiple biological processes including midline and tracheal development, circadian rhythms, and for the activation of gene transcription in response to environmental toxins [66,68]. More specific, Sim and Trh control development of the central nervous system midline and the trachea, respectively [69,70,71]. Clk/ARNT heterodiner activates a feedback loop control the persistence and period of circadian rhythms [72,73]. It is known that NlMet mediates JH signal pathway and plays a role in the ovariole development and egg maturation of the brown planthopper [24], and it could likely be involved in resistance to insecticides [74,75].

There is only one member of NlbHLHs for group D, namely NlEmc. Group D proteins, which include Id, extra macrochaetae (Emc), Heira, and Hhl462, are unable to bind DNA due to lack of a basic domain. They act as antagonists of group A proteins [19,76].

There are eight members of NlbHLHs (NlHey, NlStich1, NlSide, NlDpn, NlH, NlE(spl)1, NlE(spl)2, NlE(spl)3) in group E. Group E proteins are formed by WRPW-bHLH proteins such as Hairy and Enhancer of Split that preferentially bind to sequences referred as N boxes (CACGGC or CACGAC). They have only low affinity for E-boxes, and possess a Pro instead of an Arg residue at a crucial position in the bHLH domain [77]. These proteins usually contain two characteristic domains named “Orange” and “WRPW” peptide in the carboxyl terminus, and mainly regulate embryonic segmentation, somitogenesis and organogenesis. It is notable that NlE(spl)3 lacks the two characteristic domains, suggesting functional defects of this protein.

Group F proteins have the COE domain, which has an additional domain involved in dimerization and DNA binding, that are divergent in sequence from the other groups described. It has only one family (Knot/Collier), and mainly regulates head development and formation of olfactory sensory neurons [20,78,79]. Two members of NlbHLHs([NlKn(col)1 and NlKn(col)2) are in this group. However, NlKn(col)2 lacks the COE domain, suggesting functional defects of this protein.

### 3.6. The bHLH Repertoire of *N. lugens* and Other Insect Species

This study characterized the orthologs of the 60 *NlbHLH*s. Thus far, the *bHLH* members from 11 insect species are available and listed in Table 3. The total number of identified *NlbHLH*s (60) is comparable with 54, 48, 57, 51, 50, 49, 52, 59, 55, 55, 57 and 55 *bHLH* members in *A. pisum*, *N. vitripennis*, *H. saltator*, *A. mellifera*, *T. castaneum*, *L. decemlineata*, *B. mori*, *D. melanogaster*, *A. aegypti*, *A. gambiae*, *C. quinquefasciatus* and *P. humanus corporis*, respectively. It can be seen that all of the studied insect species lack genes of families Oligo, MyoRb and Figα, suggesting these hallmark members in other organisms may have no role in insects. All examined insect species such far have only one member in 10 bHLH families including E12/E47, Beta3, Hand, SCL, NSCL, SRC, Myc, ARNT, Trh and HIF, except for *C. quinquefasciatus* with two *Trh* members. Members of MyoD, Net, Paraxis, Mad or MLX are missing in some insect species. Nevertheless, the comparable number of bHLH families and similar orthologs found among insects strongly suggest that the set of *NlbHLH* we retrieved is likely to be almost complete, hence represents an accurate view of the *bHLH* repertoire of planthoppers. In addition to the total number of genes, another obvious difference is the discrepancy of H/E(spl) family members. *D. melanogaster* have 11 to 12 while other insects have only 4 to 8. One can also notice that *N. lugens* has one or two more genes in family Ngn, Delilah, SREBP, Sim and COE than most of the insect species. On the other hand, we failed to discover a *N. lugens* gene in the ASCb family, in which *A. pisum* has one. Furthermore, similar to *H. saltator* bHLHs, thirteen NlbHLH families have more than one member (accounted for about 29% of all the families), while in most other insects, families with more than one member are fewer (range of 13% to 20% with an average of 16%). This suggests that some of the *N. lugens bHLH* genes were originated through duplications. The divergence of *bHLH*s in insects suggests that those members may play different roles due to adaptations of specific biological niches.

## 4. Conclusions

The bHLH proteins play pivotal roles in a wide variety of biological processes. In this study, 60 *bHLH*s encoded in *N. lugens* genome were identified. Through multiple sequence alignment and ingroup phylogenetic analysis using *bHLH*s identified from *D. melanogaster* and *A. pisum*, all 60 *NlbHLH*s have been successfully classified to bHLH groups A–F. *N. lugens* has members in all six bHLH groups. The ortholog analysis and domain prediction revealed that NlTrh, NlTgo, NlClk, NlCyc and NlMet are highly conserved implying regulatory functions of many physiological processes as in other insects. In contrast, *N. lugens* specific gene duplications of *SREBP*, *Kn(col)*, *Tap, Delilah*, *Sim*, *Ato* and *Crp* suggest functional divergence. All of the results provide a foundation for further investigations of bHLH protein functions in *N. lugens* specifically, and in insects in general.

## Figures and Tables

**Table 1:** The primers used in reverse transcription polymerase chain reaction (RT-PCR) for *Nilaparvata lugens* basic helix-loop-helix (*NlbHLHs)*.

Gene Name	Forward Primer (5′to 3′)	Reverse Primer (5′to 3′)	Amplicon Size (bp)
*NlAse1*	CGTCATTCGCACTCGAGATGG	GGACATGGGCTGAACGTGGT	473
*NlAse2*	TAACAAGCCCTCACGGAGCGT	GTGTACCTTGCGTTCCAGGA	624
*NlDa*	AAGTTGAGTTCTCAGCCACGGA	GGGCACTAACTAGTCGAGTGG	735
*NlTap1*	TCACCGTCTGCATCGGACAG	CCATAGTAGGTCAACTCTTGGTG	433
*NlTap2*	ATGTAACCGTCTGCATCGGA	CCACGTAATCAGGCGAACTC	405
*NlMistr1*	GTCAAGTATGAGTGCCGACAG	CCTTGTTCCTCGTAGGGCGA	246
*NlMistr2*	CGCCAAACGGAAATGTCTGC	GTGCCATAATGTAGTTCTTGGCT	233
*NlOli*	CTACAACAGTTGAGCGGACC	GGAATCGACATCGTTCCTTGAGC	314
*NlCato*	ACCGTCGTCAAGAAGCGT	ACGCTGCAGCATATCACAC	189
*NlAto1*	AGTCGCCTCCACCGTTCTGCAA	ACTGTAGCAAGTCGTAGAGGGCA	615
*NlAto2*	AGTCGCCTCCACCGTTCTGCAA	ACTGTAGCAAGTCGTAGAGGGCA	615
*NlSage*	GATAAATTGCCGCTATGCAAGC	TCAATGGTTGTAAGCAGTGGTG	321
*NlPxs*	ATTTCAGTGTGAATTCGGCAT	AAGAACATTAACCTGTTGTGAGTTG	155
*NlTwi*	GGCAAACACGACTTGACCAG	GGCGTTCTCTTACATTCGCCA	325
*NlFer1*	AGGCACTTCCTGGATGGCTACGT	GGGTCCACACCTTGGCGTACAT	586
*NlFer2*	AGAATGCAGTACAAGCGGTC	CATCCTTCTGTTCTGAATGTGC	379
*NlHand*	AACGAGGTGCCTGTCATACG	TTTACCTGATTGGTGGCCCT	302
*NlSCL*	TGCTGAGGAAGGTGTTCACC	GCGTCGGATGCGTTACTCAA	517
*NlDel1*	AAGCTACTCGTTGCGACCCAG	ACTCTGAAATGAGGCTGACGT	602
*NlDel2*	TAACTCCGATTTGGCGTCGACC	TCAAAATCCACCGCTGACGT	249
*NlDel3*	CCATGAACCCGCAGGTGCTA	GGGCGTCAACTTGTAGGGCT	399
*NlMnt*	TGAGATTAGGAACTCGCGAAGTG	CAATGAGTAGAATTGGAAGGGCT	703
*NlMad*	AATGGTTCCCCTGGGCAACGA	GAGTCGGCTGGTGGACATAGC	505
*NlDm*	GTATGAACCGCGACTGGCTCCA	AGACTGCGGGCCACCGTCTT	547
*NlUSF*	TAATTCCTGATTGCGCTCAGGAC	GATCTGAATTGGGTATGATGCCA	252
*NlCrp2*	CCGAATACCACATTCACTCG	ATCACTGAGCCAGGGTATGG	248
*NlBmx*	CCATCAAGAAGGGGTATGACTCG	GCCAACTGAAGACACATGCT	378
*NlMlx*	ACAAATCCTACTGGCAGCGA	TGATATGCACAGCTGCCGAG	1176
*NlSREBP3*	GCAGATGGCCGGTCAACCTT	GTCCTTGGATCGCCTTTGCAG	1207
*NlTai*	TATGTCAGCACAGCAAGTGCCTT	ACTTCTAGGAGGAGATTGCCGAA	271
*NlDys*	AGGTTCGACACGAACAAGTC	GTAACTGCGAAAGACGCTGTC	130
*NlSim1*	ACATCGACCAAGCGGAAGTTGCA	TGACACTGGTGTATCCAGCCGTG	472
*NlSim2*	TCATCTACTCCAGACGCTGG	TGCATTCCTTTTCGCTAGGACG	283
*NlTrh*	CGTGATCGAAACTGCAAGGTTCG	TTACTTTGAGCGATTTGGCAGCT	574
*NlSima*	CACCTCGACAAGGCGTCCAT	CGTAGCCAAGAAACTCTTCCA	1384
*NlTgo*	CACTCGATGGACGGCAAGTT	TGGCTGTGCGTGGTAGAGTG	810
*NlCyc*	TACGCGATGTCTCGCAAGCTGGA	ACAATGTACTCGCGGTCCATCTG	1194
*NlMet*	TATCGGTTCCACTCCACAAA	AAGGGATCATTGTTGAAGCC	440
*NlEmc*	TGTGACTTGCAGTACGCTCTGGA	AGGCTTCCTGCGTGGAACAC	196
*NlHey*	TGGACTACCACAACATCGGCTT	TTCATCTGAGAGGAAACCTGGT	607
*NlSide*	GAGGACATGCTGATGGCCGTCAA	AGACATCTTCGTCCTTGTCGGCA	568
*NlDpn*	TACCTGGAAACGTTCTGCCAT	TGTTTCAGTAGATGGTTGAGGCT	554
*NlH*	AACTGGAGAAAGCGGACATCCTT	TGGGGATAGTGCCTCAACAA	1681
*NlE(spl)1*	GAAGGCTGACATCCTCGAGC	CTACCACGGCCTCCAGACTG	482
*NlE(spl)3*	GGAAAGCGACGAGGATTACTG	GCGATTTGAGTTCATTGAGGCA	191
*NlKn(col)1*	TTGATCCCTCAGATGGCCTGTA	GCAAAACTGTTTCGACTTGTAGG	266
*NlKn(col)2*	TATGTCTCCCTGAACGAGCCA	TATTTGGAAGACCCGACCAGTGG	680

**Table 2:** A complete list of basic helix-loop-helix (*bHLH)* genes from *Nilaparvata lugens*.

No.	Gene Name	Family	Fruit Fly Homolog	Statistical Support				Gene ID	Evidence Support
				NJ	MP	ML	Bayesian		
01	*NlAse1*	ASCa	*ase*	99	97	87	99	NA	EST
02	*NlAse2*	ASCa	*ase*	99	100	98	68	NLU023528	RT-PCR and EST
03	*NlDa*	E12/E17	*da*	100	100	100	100	NLU002710	RT-PCR and EST
04	*NlNau*	MyoD	*nau*	99	99	95	100	NLU022422	RT-PCR and EST
05	*NlTap1*	Ngn	*tap*	97	91	91	100	NLU007911	RT-PCR and EST
06	*NlTap2*	Ngn	*tap*	97	92	91	100	NLU023195	RT-PCR and EST
07	*NlMistr1*	Mist	*Mistr*	96	89	94	100	NLU012420	RT-PCR and EST
08	*NlMistr2*	Mist	*Mistr*	100	98	98	100	NLU027753	RT-PCR and EST
09	*NlOli*	Beta3	*Oli*	100	100	100	100	NLU011046	RT-PCR and EST
10	*NlCato*	Atonal	*cato*	37	97	78	98	NLU013048	RT-PCR and EST
11	*NlAto1*	Atonal	*ato*	99	88	92	98	NLU020408	RT-PCR and EST
12	*NlAto2*	Atonal	*ato*	98	86	92	98	NLU012608	RT-PCR and EST
13	*NlNet*	Net	*net*	100	99	97	100	NLU003697	EST
14	*NlMyoR*	MyoRa	*MyoR*	99	97	95	100	NLU020439	EST
15	*NlSage*	Mesp	*sage*	100	100	96	100	NLU017450	RT-PCR and EST
16	*NlPxs*	Paraxis	*Pxs*	88	77	80	100	NA	RT-PCR and EST
17	*NlTwi*	Twist	*twi*	98	94	79	100	NLU023739	RT-PCR and EST
18	*NlFer1*	PTFa	*Fer1*	99	92	72	98	NLU018740	RT-PCR and EST
19	*NlFer2*	PTFb	*Fer2*	99	94	65	92	NLU001388	RT-PCR and EST
20	*NlHand*	Hand	*Hand*	98	93	65	97	NLU005290	RT-PCR and EST
21	*NlSCL*	SCL	*SCL*	100	100	99	100	NLU016321	RT-PCR and EST
22	*NlNSCL*	NSCL	*NSCL*	100	99	95	100	NLU009115	EST
23	*NlDel1*	Delilah	*del*	96	91	77	96	NLU025535	RT-PCR and EST
24	*NlDel2*	Delilah	*del*	94	87	78	93	NLU027494	RT-PCR and EST
25	*NlDel3*	Delilah	*del*	94	90	77	95	NLU005401	RT-PCR and EST
26	*NlMnt*	Mnt	*Mnt*	96	88	90	100	NLU002070	RT-PCR and EST
27	NlMad*	Mnt	*ApMad*	100	100	100	100	NLU010490	RT-PCR and EST
28	*NlMax*	Max	*Max*	99	97	97	100	NA	EST
29	*NlDm*	Myc	*dm*	81	82	97	100	NLU025779	RT-PCR and EST
30	*NlUSF*	USF	*USF*	91	68	94	100	NLU023467	RT-PCR and EST
31	*NlMitif*	MITF	*Mitif*	100	100	100	100	NLU017474	EST
32	*NlCrp1*	AP4	*Crp*	83	44	50	88	NLU016559	EST
33	*NlCrp2*	AP4	*Crp*	98	96	88	100	NLU011530	RT-PCR and EST
34	*NlBmx*	TF4	*bmx*	99	81	98	95	NA	RT-PCR and EST
35	*NlMlx*	MLX	*MLX*	100	97	97	100	NLU009394	RT-PCR and EST
36	*NlSREBP1*	SREBP	*SREBP*	94	73	81	100	NLU005608	EST
37	*NlSREBP2*	SREBP	*SREBP*	96	73	81	100	NLU006435	EST
38	*NlSREBP3*	SREBP	*SREBP*	90	59	72	98	NLU021448	RT-PCR and EST
39	*NlTai*	SRC	*tai*	93	99	100	100	NLU023056	RT-PCR and EST
40	*NlClk*	Clock	*clk*	100	100	98	100	NLU027428	EST
41	*NlDys*	AHR	*dys*	100	100	100	100	NA	RT-PCR and EST
42	*NlSs*	AHR	*ss*	100	100	100	100	NLU022623	EST
43	*NlSim1*	Sim	*sim*	87	79	71	78	NLU022755	RT-PCR and EST
44	*NlSim2*	Sim	*sim*	93	83	72	74	NLU008712	RT-PCR and EST
45	*NlTrh*	Trh	*trh*	99	89	96	84	NLU009957	RT-PCR and EST
46	*NlSima*	HIF	*sima*	79	87	96	100	NLU019462	RT-PCR and EST
47	*NlTgo*	ARNT	*tgo*	100	100	100	100	NLU026318	RT-PCR and EST
48	*NlCyc*	Bmal	*cyc*	97	88	55	86	NA	RT-PCR and EST
49	*NlMet*	Met	*Met*	77	68	77	95	NA	RT-PCR and EST
50	*NlEmc*	Emc	*emc*	93	92	88	100	NLU011228	RT-PCR and EST
51	*NlHey*	Hey	*Hey*	96	89	84	92	NLU027503	RT-PCR and EST
52	*NlStich1*	Hey	*Stich1*	100	100	100	100	NLU010132	EST
53	*NlSide*	H/E(spl)	*side*	97	89	95	100	NLU019226	RT-PCR and EST
54	*NlDpn*	H/E(spl)	*dpn*	61	n/m	21	n/m	NLU021732	RT-PCR and EST
55	*NlH* *	H/E(spl)	?-*ApH*	93	93	55	67	NLU017783	RT-PCR and EST
56	*NlE(spl)1* *	H/E(spl)	?-*ApHES1*	93	65	62	62	NLU012936	RT-PCR and EST
57	*NlE(spl)2* *	H/E(spl)	*?*	n/m	n/m	n/m	n/m	NLU007850	EST
58	*NlE(spl)3* *	H/E(spl)	?-*ApHES1*	99	96	85	89	NLU021733	RT-PCR and EST
59	*NlKn(col)1*	COE	*Kn(col)*	100	100	100	100	NLU001955	RT-PCR and EST
60	*NlKn(col)2*	COE	*Kn(col)*	100	100	100	100	NLU011325	RT-PCR and EST

*NlbHLH* genes were named according to their *D. melanogaster* homologs. Bootstrap values were obtained from in-group phylogenetic analyses with *D. melanogaster* or *A. pisum bHLH* motif sequences using neighbor-joining (NJ), maximum parsimony (MP), maximum-likelihood (ML) and Bayesian algorithms, respectively. OsRa (the rice *bHLH* motif sequence of R family) was used as outgroup in each constructed tree. n/m means that a *N. lugens bHLH* does not form a monophyletic group with any other single *bHLH* motif sequence. * means that orthology of the gene was defined through in-group phylogenetic analyses with *bHLH* orthologs from *A. pisum*. RT-PCR, Reverse Transcription-Polymerase Chain Reaction; EST, Expressed Sequence Tag.

**Table 3:** Comparisons of bHLH family members from twelve insect species.

Group	Family name	N.l.	A.p.	N.v.	H.s.	A.m.	T.c.	L.d.	B.m.	D.m.	A.a.	A.g.	C.q.	P.h.
A	ASCa	2	0	2	2	2	3	1	4	4	4	2	4	2
A	ASCb	0	1	0	0	0	0	0	0	0	0	0	0	1
A	MyoD	1	0	1	1	1	1	1	1	1	1	1	1	1
A	E12/E17	1	1	1	1	1	1	1	1	1	1	1	1	1
A	Ngn	2	1	1	1	1	1	0	1	1	1	2	2	1
A	NeuroD	0	0	0	0	0	1	0	0	0	1	0	0	0
A	Atonal	3	3	3	3	3	3	2	1	3	5	4	5	3
A	Mist	2	2	2	2	2	1	1	1	1	1	1	1	2
A	Beta3	1	1	1	1	1	1	1	1	1	1	1	1	1
A	Oligo	0	0	0	0	0	0	0	0	0	0	0	0	0
A	Net	1	1	0	1	1	1	1	1	1	1	1	1	1
A	Delilah	3	1	0	0	0	2	1	1	1	1	1	1	1
A	Mesp	1	1	1	1	1	0	0	1	1	1	1	1	1
A	Twist	1	1	1	2	1	1	1	1	1	1	1	1	1
A	Paraxis	1	1	1	1	1	1	1	1	1	1	1	1	0
A	MyoRa	1	1	0	1	1	1	1	1	1	1	1	1	1
A	MyoRb	0	0	0	0	0	0	0	0	0	0	0	0	0
A	Hand	1	1	1	1	1	1	1	1	1	1	1	1	1
A	PTFa	1	1	0	1	1	1	1	1	1	1	2	1	1
A	PTFb	1	2	2	2	1	2	1	1	2	2	2	2	2
A	SCL	1	1	1	1	1	1	1	1	1	1	1	1	1
A	NSCL	1	1	1	1	1	1	1	1	1	1	1	1	1
B	SRC	1	1	1	1	1	1	1	1	1	1	1	1	1
B	Figα	0	0	0	0	0	0	0	0	0	0	0	0	0
B	Myc	1	1	1	1	1	1	1	1	1	1	1	1	1
B	Mad	1	1	1	0	0	1	1	0	0	0	0	0	0
B	Mnt	1	1	1	2	1	1	1	1	1	1	1	1	1
B	Max	1	3	1	2	1	1	1	1	1	1	1	1	1
B	USF	1	1	1	2	2	1	1	1	1	1	1	1	1
B	MITF	1	0	1	1	1	1	1	1	1	1	1	1	2
B	SREBP	3	1	1	1	1	1	1	1	1	1	1	2	1
B	AP4	2	1	2	2	1	1	1	1	1	1	1	1	1
B	MLX	1	1	1	1	1	0	1	1	1	1	1	1	1
B	TF4	1	2	1	1	1	1	1	1	1	1	1	1	1
C	Clock	2	2	2	2	2	2	2	3	3	2	2	2	2
C	ARNT	1	1	1	1	1	1	1	1	1	1	1	1	1
C	Bmal	1	1	1	1	1	1	1	2	1	1	1	1	1
C	AHR	2	2	2	3	2	1	2	3	2	2	2	2	2
C	Sim	2	1	1	1	1	0	1	1	1	1	2	1	1
C	Trh	1	1	1	1	1	1	1	1	1	1	1	2	1
C	HIF	1	1	1	1	1	1	1	1	1	1	1	1	1
D	Emc	1	1	1	1	1	1	1	1	1	1	2	1	1
E	Hey	2	3	2	2	2	2	2	2	2	3	3	3	2
E	H/E(spl)	6	6	4	6	6	6	8	5	11	4	4	4	8
F	COE	2	1	1	1	1	1	1	1	1	1	1	1	1
		60	54	48	57	51	50	49	52	59	55	55	57	55

The *bHLHs* are from N.l. (Nilaparvata lugens); A.p. (*Acyrthosiphon pisum*) [7]; N.v. (*Nasonia vitripennis*) [8]; H.s. (*Harpegnathos saltator*) [9]; A.m. (*Apis mellifera*) [10]; T.c. (*Tribolium castaneum*) [11]; L.d. (*Leptinotarsa decemlineata*) [12]; B.m. (*Bombyx mori*) [13]; D.m. (*Drosophila melanogaster*) [16]; A.a. (*Aedes aegypti*) [14]; A.g. (*Anopheles gambiae*) [14] and C.q.(*Culex quinquefasciatus*) [14]; P.h. (*Pediculus humanus corporis*).

## Supplementary Materials

The following are available online at www.mdpi.com/2073-4425/7/11/100/s1, Supplementary figure 1: The NJ (A), MP (B) and Bayesian (C) trees of 60 *Nl*bHLH members with 59 *D. melanogaster* bHLH members. These trees summarize the evolutionary relationship between the *NlbHLH*s and *DmbHLH*s, which were rooted using *OsRa* (a rice bHLH motif sequence of R family) as outgroup. The trees are based on a multiple alignment that includes 59 *DmbHLH* and 60 *NlbHLH* members. For simplicity, branch lengths of the trees are not proportional to distances between sequences; Supplementary file 2: The nucleotide sequences of 60 *NlbHLHs*.

## Abbreviations

Nl: *Nilaparvata lugens*; bHLH: Basic helix-loop-helix; NlbHLH: *Nilaparvata lugens* bHLH; Ap: *Acyrthosiphon pisum*; Dm: *Drosophila melanogaster*; RNAi: RNA interference; ML: Maximum-likelihood; NJ: Neighbor-joining; MP: maximum parsimony; nr: non-redundant database; bp: base pairs; TE: Transposable element; PCR, polymerase chain reaction; SMART: Simple Modular Architecture Research Tool; CDART: Conserved Domain Architecture Retrieval Tool.
