# Supplementary material for: A Genome-Wide Identification and Analysis of the Basic Helix-Loop-Helix Transcription Factors in Brown Planthopper, Nilaparvata lugens
Source: Genes (Basel). 2016 Nov 18;7(11):100. doi: 10.3390/genes7110100 (PMC5126786; doi:10.3390/genes7110100)
Supplement: Supplementary file 1 [file genes-07-00100-s001.docx]

Supplementary Materials: A Genome-Wide Identification and Analysis of the Basic
Helix-Loop-Helix Transcription Factors in Brown Planthopper, *Nilaparvata lugens*

Pin-Jun Wan, San-Yue Yuan, Wei-Xia Wang, Xu Chen, Feng-Xiang Lai and Qiang Fu


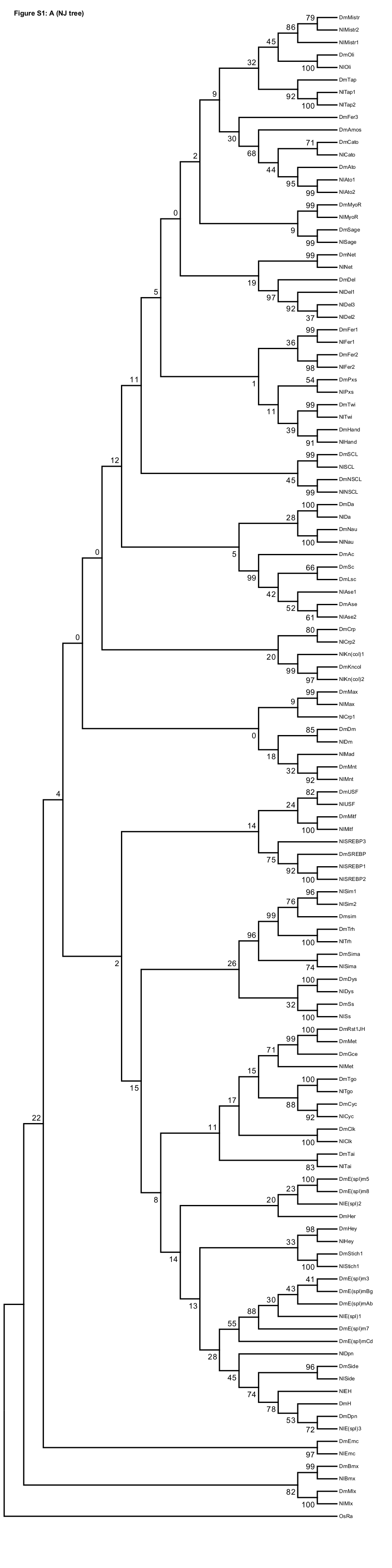


**Figure S1.** A (Neighbor-joining (NJ) tree).


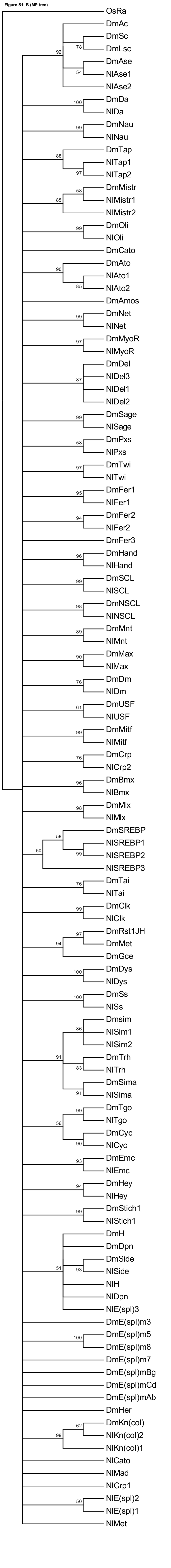


**Figure S1.** B (Maximum parsimony (MP) tree).





**Figure S1.** C (Bayesian tree).
